# Commentary: Systemic effects of IL-17 in inflammatory arthritis

**DOI:** 10.3389/fcvm.2019.00183

**Published:** 2019-12-10

**Authors:** Pietro Enea Lazzerini, Franco Laghi-Pasini, Mohamed Boutjdir, Pier Leopoldo Capecchi

**Affiliations:** ^1^Department of Medical Sciences, Surgery and Neurosciences, University of Siena, Siena, Italy; ^2^VA New York Harbor Healthcare System, SUNY Downstate Medical Center, New York, NY, United States; ^3^NYU School of Medicine, New York, NY, United States

**Keywords:** IL-17, cardiac arrhythmias, inflammatory arthritis, structural remodeling, electric remodeling, connexin43

The recent article by Beringer and Miossec (1) provided a detailed examination of the pleiotropic effects of interleukin-17 (IL-17), highlighting their potential role in promoting systemic co-morbidities in inflammatory arthritis (IA). In particular, given the increased cardiovascular risk characterizing these patients, Beringer and Miossec extensively discussed how the effects of IL-17 on blood vessels and heart might accelerate atherosclerosis and related complications, as well as hypertension and cardiomyopathy development (1).

However, the authors did not mention a number of recent studies suggesting a significant impact of IL-17 on the arrhythmic risk. This aspect should be emphasized as cardiac arrhythmias, particularly ventricular arrhythmias (VA) and cardiac arrest, atrial fibrillation (AF) and conduction disturbances, are more commonly observed in IA than in the general population, significantly contributing to morbidity and mortality (2–5). Although the underlying mechanisms are probably complex, increasing evidence points to a key role for systemic inflammation, at least in part via direct effects of cytokines, specifically TNFα, IL-6 and IL-1, able to induce cardiac remodeling both structural (damage/fibrosis promoting re-entry mechanisms) (2), and electric by modulating the expression/function of specific ion channels in the cardiomyocyte (*inflammatory cardiac channelopathies*) (6, 7). Such channels also include gap-junctions, intercellular channels mediating electrical coupling between two adjacent cardiomyocytes, formed by proteins named connexins (Cxs). Among different connexins, Cx43 is ubiquitously expressed in the heart where critically contributes to impulse conduction velocity and refractoriness heterogeneity in ventricles, atria and atrio-ventricular (AV) junction (8–10). Evidence indicates that TNFα, IL-6, and IL-1 can promote arrhythmias by inhibiting cardiac Cx43 expression (6, 7, 11). In this scenario, IL-17 might play an important additional role.

By using the Langendorff perfusion model, Chang et al. (12) demonstrated that acute administration of IL-17 can induce VA in rabbit hearts, along with decreasing conduction velocity and prolonging action potential duration, all these changes being prevented by perfusion with an anti-IL-17 neutralizing antibody. The same authors demonstrated that VAs inducibility was also significantly increased in a rabbit model of ischemic heart failure following chronic intravenous administration of IL-17. In the left ventricle of these animals, collagen production, fibrosis and apoptosis were markedly enhanced (12). Moreover, in rats with myocardial infarction, reduced IL-17 expression in the myocardium was associated with increased Cx43 expression, and lower susceptibility to VAs induction upon programmed electrical stimulation (13). Furthermore, the group of Saffitz showed the implication of IL-17 in disruption of desmosomal proteins, i.e., translocation of plakoglobin from cell-cell junction resulting in granulomatous myocarditis as potential pathogenic links to arrhythmogenic right ventricular cardiomyopathy (ARVC) (14). Notably, in ARVC, where Cx43 expression has been reported to be reduced (15, 16), myocardial IL-17 level is increased. Interleukin-17A levels are also elevated in patients with AF (17), and treatment with anti-IL-17A monoclonal antibody markedly suppressed AF development in a rat model of sterile pericarditis, concomitantly reducing atrial inflammation and fibrosis (18). Finally, a recent genome-wide association study identified a single-nucleotide-polymorphism in the gene encoding IL-17D as a key determinant of electric conduction in the AV node (19). This finding intriguingly suggests a pathogenic role for IL-17 in AV disturbances observed in IA, possibly by modulating Cx43 expression on myocytes and/or macrophages in the AV node (9).

Altogether, these data point to a significant involvement of IL-17 in arrhythmogenesis (Figure 1). Further research is warranted to better dissect its specific role in cardiac electrophysiology, as well as the potential beneficial effects of IL-17 targeted therapies on arrhythmic disorders in IA. In this regard, two anti-IL-17 agents are currently approved for IA (specifically psoriatic arthritis), i.e., secukinumab and ixekizumab (20). However, although numerous randomized controlled trials demonstrated the cardiovascular safety of these drugs (20, 21), to date no specific information is available on their impact on arrhythmic events in IA patients.

**Figure 1:**
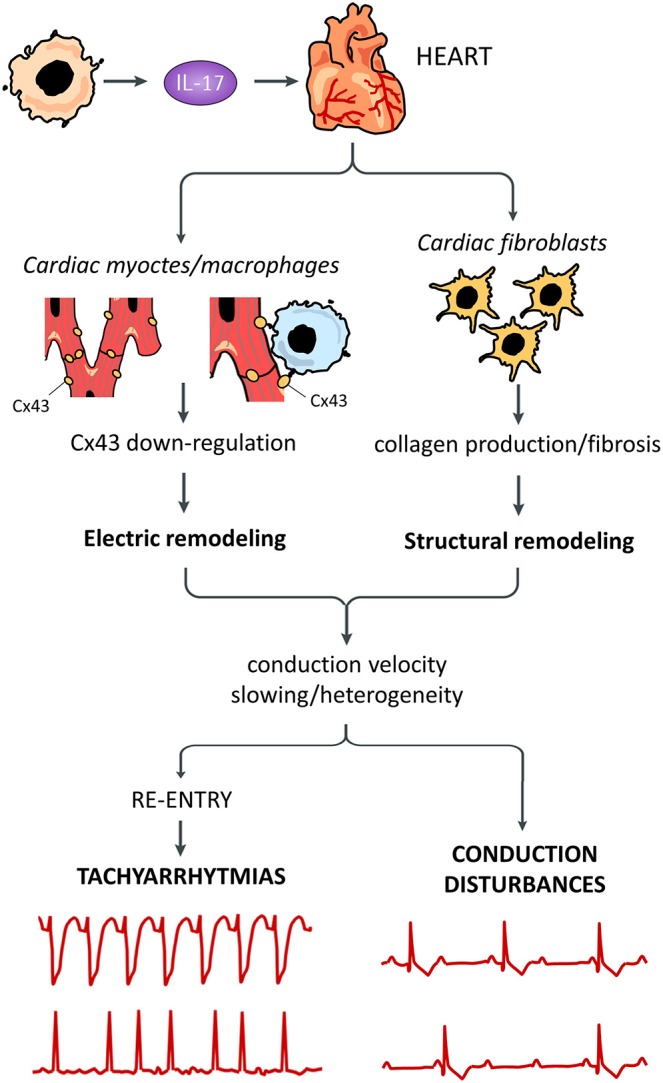
Putative pro-arrhythmic effects of IL-17. Systemically released IL-17 can promote arrhythmogenesis by affecting different cells in the heart. Cardiac fibroblast are stimulated by IL-17 to produce high amounts of collagen with tissue fibrosis, resulting in structural remodeling. IL-17 can also induce electric remodeling by down-regulating connexin43 (Cx43) expression, possibly in both cardiomyocytes and cardiac macrophages. Remodeling phenomena are responsible for decrease/heterogeneity of electric impulse conduction velocity throughout the working and conducting myocardium, in turn promoting re-entry-driven tachyarrhythmias and conduction disturbances.

## Author Contributions

PL: conception, design, and drafting of the work. FL-P, MB, and PC: revising the draft of the work critically for important intellectual content and agreement to be accountable for all aspects of the work in ensuring that questions related to the accuracy or integrity of any part of the work are appropriately investigated and resolved. PL, FL-P, MB, and PC: final approval of the version to be published.

### Conflict of Interest

The authors declare that the research was conducted in the absence of any commercial or financial relationships that could be construed as a potential conflict of interest.
